# Heart meets brain: A 25‐year‐old with a constellation of neurologic symptoms and heart failure

**DOI:** 10.1002/acn3.52196

**Published:** 2024-10-23

**Authors:** Noellie Rivera Torres, Rohini Samudralwar, Joseph Berger

**Affiliations:** ^1^ Neurology Department University of Pennsylvania Philadelphia Pennsylvania USA

interACTN Case #42: Available: https://interactn.org/2024/08/21/case‐42‐heart‐mets‐brain‐a‐25‐year‐old‐with‐a‐constellation‐of‐neurologic‐symptoms‐and‐heart‐failure/

## Summary of Case

A 25‐year‐old previously healthy female presented with headache, nausea, R facial droop, dysarthria, unsteady gait, and worsening shortness of breath (SOB). Ten days prior, she had presented to an outside hospital emergency department (ED) with intermittent numbness in both upper extremities, associated chest pressure, and shortness of breath of 4 days' duration. The numbness had progressed more than 7 days to involve her chest, trunk, and legs with lack of sensation while urinating and defecating. She gradually noticed dysarthria and gait instability and started dropping objects. While being evaluated at the ED, she developed acute respiratory failure with cardiogenic shock requiring intubation. On neurologic examination, she awoke to voice and shook her head yes/no inappropriately. Direction changing nystagmus was present in primary gaze and with eye movements. Strength was at least antigravity in all extremities with brisk lower extremity reflexes and no pathological reflexes.

**Figure 1:**
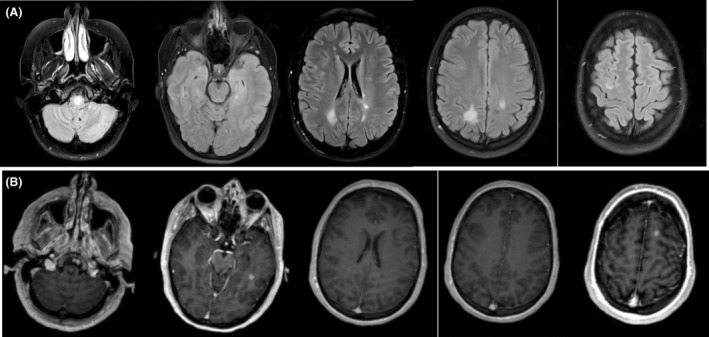
MRI brain with and without contrast on admission: (A) T2 FLAIR sequence showing hyperintensitiesy in the ventral medulla, central pons, and confluent periventricular and cortical hyperintensities. (B) T1 post‐contrast sequence showing enhancement of the medullary lesion, left inferior temporal lobe, and left frontal lobe.

Brain MRI with and without contrast (Fig. 1) demonstrated T2 FLAIR hyperintensities in the ventral medulla, central pons, periventricular white matter, and cortex. T1 post‐contrast sequences revealed enhancement of the medullary lesion, left inferior temporal lobe, and left frontal lobe. Lumbar puncture revealed six CSF‐unique oligoclonal bands. Serologic labs were negative for aquaporin‐4 and myelin oligodendrocyte glycoprotein (MOG) antibodies, infectious and systemic inflammatory sources. Cardiac enzymes were significantly elevated and uprising with the highest at 10,907 uIU/mL. Echocardiogram showed an estimated ejection fraction of 20–25%, severe global hypokinesis, and mild LV dilation. Cardiac catheterization ruled out coronary atherosclerosis.

**Figure 2:**
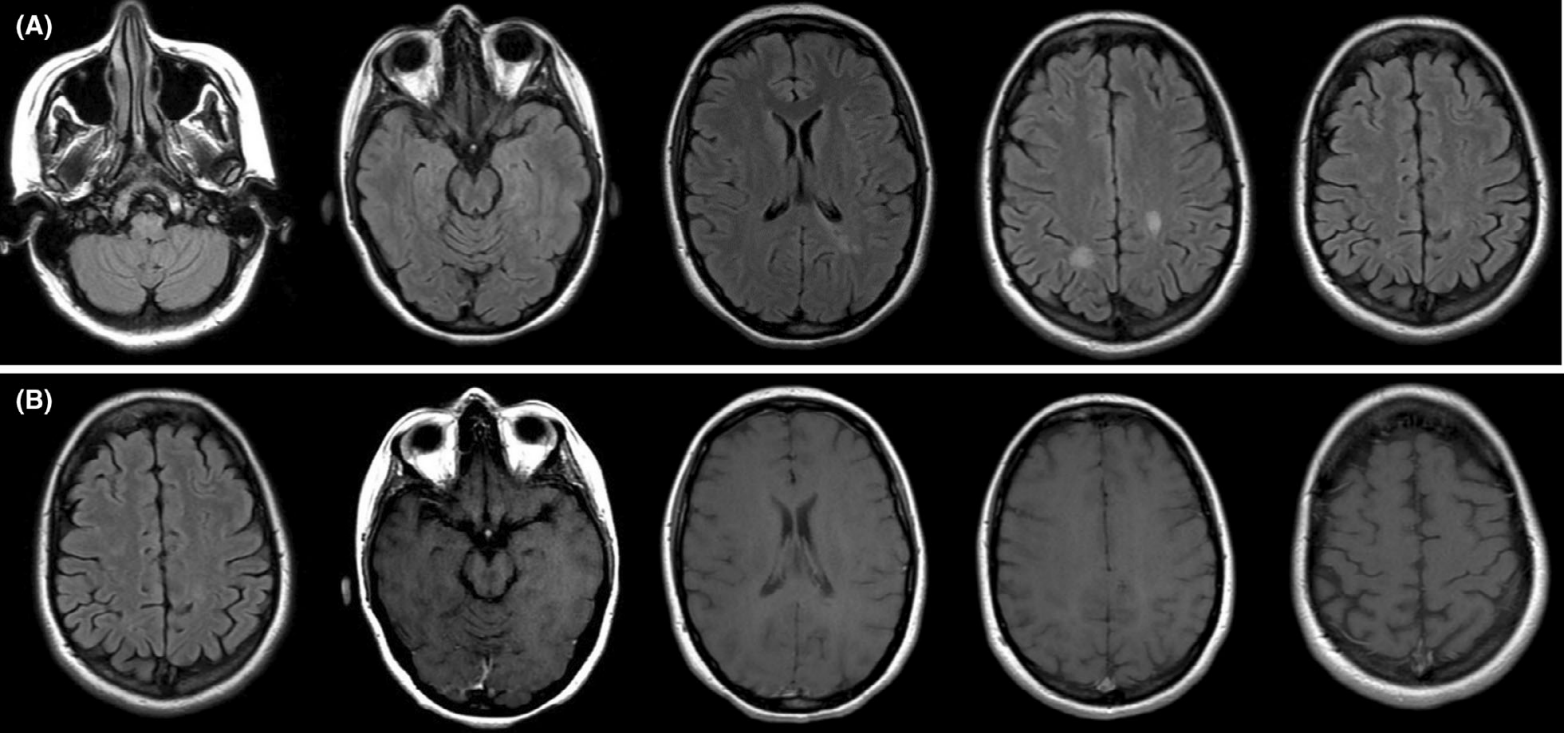
MRI brain with and without contrast 4 months after admission: (A) T2 FLAIR sequence showing improved hyperintensities in the medulla and periventricular white matter. (B) T1 post‐contrast sequence showing resolution of enhancing lesions. No new lesions seen.

This patient met the 2017 McDonald's criteria for Multiple Sclerosis and the criteria for Takotsubo Cardiomyopathy.^1^, ^2^ Although uncommon, fulminant demyelination presenting as Takotsubo cardiomyopathy in MS can occur, particularly in association with acute medulla oblongata lesions.^3^, ^4^ After adequate recognition, the patient was treated with intravenous steroids and plasmapheresis with significant clinical and radiological improvement (Fig. 2).^5^

## Diagnosis

Multiple sclerosis (MS) and acute Takotsubo cardiomyopathy secondary to an acute medullary lesion.

### Take‐Home Points

- Takotsubo cardiomyopathy can be seen as a fulminant presentation of multiple sclerosis relapse secondary to an acute medullary lesion.
- It is important to recognize this presentation to treat acutely with steroids and PLEX if not responding initially to steroids.
- Multiple sclerosis treatment involves acute treatment, disease‐modifying therapy, and symptomatic treatment and support.
- Treatment of Takotsubo cardiomyopathy secondary to acute demyelination is aimed at treating the acute inflammation secondary to the demyelinating disease and symptomatic management.

## Author Contributions

Dr. Rivera Torres was involved in the development, writing, editing, and submission of the article. Dr. Samudralwar and Dr. Berger wereinvolved in the development and editing of the article.

## Funding Information

No funding sources to report.
